# Gene expression changes following extinction testing in a heroin behavioral incubation model

**DOI:** 10.1186/1471-2202-10-95

**Published:** 2009-08-07

**Authors:** Kara L Kuntz-Melcavage, Robert M Brucklacher, Patricia S Grigson, Willard M Freeman, Kent E Vrana

**Affiliations:** 1 Department of Pharmacology, Pennsylvania State University College of Medicine, Hershey, PA, USA; 2 Functional Genomics Core Facility, Pennsylvania State University College of Medicine, Hershey, PA, USA; 3 Department of Neural and Behavioral Sciences, Pennsylvania State University College of Medicine, Hershey, PA, USA

## Abstract

**Background:**

A number of gene expression studies have investigated changes induced by drug exposure, but few reports describe changes that persist following relapse. In this study, genome-wide analysis of gene expression was conducted following an extinction session (90 min) in rats that expressed behavioral incubation of heroin-seeking and goal-directed behavior. As an important modulator of goal-directed behavior, the medial prefrontal cortex (mPFC) was the target of genomic analysis. Rats were trained to self-administer heroin during 3 h daily sessions for 14 d. Following the self-administration period, rats were reintroduced to the self-administration chambers for a 90-minute extinction session in which they could seek heroin, but received none. Extinction sessions were conducted on groups after either 1 d or 14 d of drug-free enforced abstinence to demonstrate behavioral incubation.

**Results:**

Behavioral data demonstrated incubation (increased expression) of heroin-seeking and goal-directed behavior after the 14 d abstinent period. That is, following 14 d of enforced abstinence, animals displayed heightened drug-seeking behavior when returned to the environment where they had previously received heroin. This increased drug-seeking took place despite the fact that they received no drug during this extinction session. Whole genome gene expression analysis was performed and results were confirmed by quantitative real-time PCR (RT-qPCR). Microarrays identified 66 genes whose expression was identified as changed by at least 1.4 fold (p < 0.02) following 14 d of abstinence and the 90-minute extinction session compared to the saline treated controls. Orthogonal confirmation by RT-qPCR demonstrated significant alterations in *bdnf*, *calb1*, *dusp5*, *dusp6*, *egr1*, *npy*, *rgs2*.

**Conclusion:**

Ontological analysis indicates that several of the genes confirmed to be changed are important for neuroplasticity, and through that role may impact learning and behavior. The importance of drug-seeking behavior and memory of previous drug-taking sessions suggest that such genes may be important for relapse. The global gene expression analysis adds to the knowledge of heroin-induced changes and further highlights similarities between heroin and other drugs of abuse.

## Background

The challenge for drug abuse treatment is maintaining abstinence despite a high propensity for abstinent patients to relapse to drug use. Although heroin has been abused for centuries, effective long-term preventions for heroin-relapse are still needed. Physiological and gene expression changes that may increase an individual's likelihood to relapse are known to exist well into a period of abstinence [1-5]. Therefore, relapse to drug use is currently being investigated on both the molecular and behavioral levels [6-10]. These studies have been aided by advances in systems biology tools, including large-scale discovery techniques such as microarrays and proteomics. These approaches are useful for discovering novel targets affected by drug abuse and examining hypotheses concerning categories of genes that are affected by drug use [11,12]. As data on gene expression following relapse accumulates, the existence of a single "relapse gene" is becoming increasingly unlikely. Therefore, macroscopic views of gene expression (pattern identification) will prove very useful for guiding research into behavioral phenomena.

Neurobiological, environmental, cue, and stress mechanisms have all been implicated in relapse to drug use. The intense craving and motivation to seek drug, reported by humans during withdrawal from drug use, is challenging to model in animals, but the need for a relapse model continues to motivate the design of new behavioral procedures. Incubation is a behavioral phenomenon that is characterized by increased drug seeking following increasing periods of abstinence after the last self-administration session [13]. Increased drug-seeking has been inferred to represent the craving that drives humans to relapse [2,14,15].

The prefrontal cortex is important for decision making and guiding behavior [16], and the medial prefrontal cortex (mPFC) is known to be especially important for goal-directed behaviors [17,18]. Because of its role in guiding goal-directed, drug-seeking behaviors, understanding the gene expression changes in this region following a period of abstinence from drug self-administration will be useful for understanding the neurobiological basis to relapse following a period of drug self-administration. Additionally, the ventral mPFC is believed to play an essential role for expression of incubation of cocaine-seeking [19] and reinstatement of cocaine-seeking [20].

In this study, we performed a whole genome analysis of gene expression in the medial prefrontal cortex of rats that displayed incubation of goal-directed behavior following 2 weeks of heroin self-administration and 2 weeks of home-cage enforced abstinence. After the abstinence period, rats (drug-naïve control animals and heroin self-administering test subjects) were reintroduced to the testing chambers for a 90-minute extinction session during which behavioral responses were recorded. This extinction session served not only to provide an opportunity to observe behavioral incubation, but also mimicked a real-life situation in which environmental cues precipitate relapse behavior following an extended period of abstinence. Following this experience of re-exposure to drug-associated context and cues, RNA was isolated from the mPFC for whole genome microarray and qPCR analyses. Ontological analyses revealed that many of the genes identified to be changed have the potential to be key components to neuroadaptations that exist at the time of relapse.

## Methods

### Heroin self-administration

**Figure 1:**
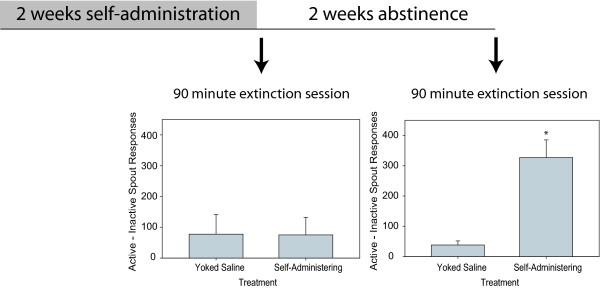
**Behavioral experimental timeline and extinction responding results**. Rats self-administered heroin for 2 weeks followed by a 90 minute extinction session that occurred either 1 day or 14 days after the final self-administration. session. Goal-directed behavior was recorded during the extinction sessions. Data represent means ± SEM. * p < 0.001.

The self-administration and extinction procedures have been previously described [21,22]. Briefly, rats that self-administered heroin displayed incubation of heroin-seeking when tested in a 90-minute extinction session that occurred after 2 weeks of enforced abstinence (see Figure 1). As described in previous work, all rats (those destined for either 1 day or 14 days of abstinence) initially underwent a habituation procedure during which they received water on a spout in the self-administration chambers. Following habituation, the rats self-administered heroin (0.06 mg/0.2 ml infusion via an in-dwelling jugular catheter) by licking the previously water-associated spout during 3 hour sessions that occurred on 14 consecutive days. Spout-licking has been the operant behavior in previous drug self-administration studies [23-25]. Two empty spouts, termed "active" and "inactive" were present in the self-administration chambers (to demonstrate that the rats focus on a goal - the heroin-associated "active" spout). A fixed ratio 10 (FR10) schedule of reinforcement existed, under which 10 consecutive responses on the active spout produced an automated injection of heroin. Self-administering rats were yoked to rats that received infusions of saline. That is, the control group was a separate set of animals that were treated exactly the same, but never received heroin (they received injections of saline anytime their matched animal received heroin).

The 14 days of heroin self-administration were followed by a 90-minute extinction session (in the same environment in which they had previously received heroin) that occurred following either 1 day or 14 days of drug-free enforced abstinence after the final self-administration session. During the extinction session, responses on active and inactive spouts were recorded, but no drug was administered. Immediately after the extinction sessions, the rats were sacrificed and brain regions were harvested. All studies were conducted in accordance with The Pennsylvania State University Institutional Animal Care and Use Committee (Protocol #2006-045), strictly adhering to the Guidelines for the Care and Use of Mammals in Neuroscience and Behavioral Research. National Research Council, 2003 National Research Council, *Guidelines for the care and use of mammals in neuroscience and behavioral research *(2003)

### Dissection and RNA isolation

#### Sacrifice and tissue dissection

Immediately following the 90-minute extinction session, all rats were sedated using Propofol (10 mg/kg, i.v.) and decapitated within 10 minutes. Brains were rapidly removed from skulls, placed in pre-chilled phosphate buffered saline (PBS) and then sectioned in an ice-chilled ASI brain slicer (ASI Instruments, Warren MI). The section from Bregma +4.4 to 2.4 mm was cut along the forceps minor (anterior to the corpus collosum) and the cortex medial to this cut was collected. This includes the cingulate (area Cg1), prelimbic cortex, infralimbic cortex, and medial orbital cortex. Following dissection, the tissue was placed in prechilled tubes, immediately frozen on dry ice, and then stored at -80°C.

#### RNA isolation

Total cellular RNA was isolated using Tri Reagent (Molecular Research Center Inc., Cincinnati, OH) [26]. Isolated RNA was further purified using an RNeasy Mini Kit for RNA clean-up (QIAGEN Sciences, Maryland). RNA quantity and quality were assessed using the RNA 6000 Nano Assay with an Agilent 2100 Bioanalyzer (Agilent, Palo Alto, CA).

### Microarray analysis

Microarray analyses were performed on samples from rats that experienced 14 d of abstinence prior to an extinction session. Studies were performed by the Penn State College of Medicine Functional Genomics Core Facility on 12 arrays (n = 6 per treatment group: 14 d abstinent self-administering and yoked saline) according to standard procedures [27]. Following the manufacturer's protocol of the Low Input Fluorescent Linear Amplification Kit (Agilent, Santa Clara, CA), 500 ng RNA with the addition of One-Color Spike Mix was denatured and incubated with T7 Promoter primer. Synthesis of cDNA followed with the addition of First-Strand buffer, DTT, dNTP mix, MMLV-RT and RNase Out and incubation at 40°C for 2 hours. Transcription of the product incorporated the Cyanine 3-CTP in the Master Mix which includes Transcription buffer, DTT, NTPs, PEG, RNase Out, pyrophosphatases and T7 RNA Polymerase, with incubation for 2 hours at 40°C. The resulting cRNA was purified using RNEasy columns (Qiagen) followed by assessment of purity, concentration and quality using a NanoDrop ND-1000 (NanoDrop Technologies, Wilmington, DE 19810) through calculated yield and Specific Activity. 1.65 μg from each sample was fragmented, denatured, and then hybridized to Agilent 4 × 44 rat whole genome microarray slides for 17 hours at 65°C. Slides were then washed according to protocol.

Microarrays were scanned with an Axon 4000B scanner with GenePix4 v4.0 software at a 5 μm resolution and 635 nm with laser power at 100%, PMT voltage at 600 V, focus position 0 μm, and lines to average = 1. Images were then imported into Agilent Feature Extraction Software. Initial quality control (positive and negative controls), exclusion of manufacturing defects (MSR spots), background subtraction was then performed and the results exported to GeneSpring GX 7.3 (Agilent Technologies). All primary array data have been deposited to Gene Expression Omnibus (accession number GSE13166).

### Microarray Data Analysis

Microarray data were normalized following import into GeneSpring GX 7.3 (Agilent Technologies) by transforming signal values less than 5.0 to an intensity of 5.0. Normalization was done per chip to the 50^th ^percentile, and per gene to the median. Values were then normalized on a per gene basis to the control group. Potential differential expression was determined with a one-way ANOVA (variances not assumed to be equal), p < 0.02 and filtered for 1.4 fold and greater differences in expression in accordance with standards for microarray analysis [28]. The use of a combination of statistical and fold-change cutoffs as opposed to traditional multiple testing corrections (*e.g*., Bonferroni) produce gene lists with the lowest rate of type I and type II errors [29]. 1.4-fold was chosen as the fold-change cutoff, as this magnitude change is at the lower range of changes we find to be confirmable by RT-qPCR. Array Data for the complete list of 66 genes that were identified to have changed expression at the p < 0.02 level of significance is included in Additional File 1. Also included are fold-changes in expression, accession numbers, and probe identification information.

### RT-qPCR analysis of gene expression

Complimentary DNA synthesis was performed on total RNA (n = 8 per treatment group: 1 d abstinent self-administering, 1 d abstinent yoked saline, 14 d abstinent self-administering and 14 d abstinent yoked saline) using Superscript III Reverse Transcriptase (Invitrogen, Carlsbad, CA). The RNA used for RT-qPCR was an aliquot from the same samples used to generate microarray probes. Comparisons at both 1 d of abstinence and 14 d of abstinence were made to gain insights into whether the expression changes observed after 14 d of abstinence had existed immediately after drug use and persisted during extended abstinence or had emerged during extended abstinence. One μg RNA, 500 ng Oligo (dT), and 10 mM each dNTP, were incubated for 5 minutes at 65°C and then chilled on ice for 2 minutes. 5× First Strand Buffer (250 mM Tris-HCL (pH8.3), 375 mM KCL, and 15 mM MgCl_2_), 5 mM DTT (final concentration), 40 U RNaseOut, and 200 U Superscript III RT were then added. The 20 μl reaction was incubated for 60 minutes at 50°C followed by a final incubation at 70°C for 15 minutes for termination. The resulting cDNA product was quantified and 20 ng of product was used in each subsequent qPCR reaction.

Quantitative PCR was carried out on a real-time detection instrument (ABI 7900HT Sequence Detection System) in 384-well optical plates using TaqMan Universal PCR Master Mix and Assay on Demand primers and probes (Applied Biosystems, Foster City, CA) as described previously [27,30]. Primer/probe sets used are listed in Table 1. SDS 2.2.2 software and the 2^-ΔΔCt ^analysis method [31] were used to quantitate relative amounts using β-actin as an endogenous control.

**Table 1:** List of genes examined in this study.

Abbreviation	Gene Name	Accession #	Assay ID
BDNF	Brain derived neurotropic factor	NM_012513	Rn02531967_s1
Ctnnb1	Catenin, beta 1	NM_053357	Rn00584431_g1
Calb1	Calbindin 1	NM_031984	Rn00583140_m1
Cdkn1b	Cyclin-dependent kinase inhibitor 1B	NM_031762	Rn00582195_m1
Chka	Choline kinase alpha	NM_017127	Rn00567492_m1
Cited2	Cbp/p300-interacting transactivator, with Glu/Asp-rich carboxy-terminal domain, 2	NM_053698	Rn00586705_m1
Crk	v-crk sarcoma virus oncogene homolog	NM_019302	Rn00467066_m1
Dusp5	Dual specificity phosphatase 5	NM_133578	Rn00592122_m1
Dusp6	Dual specificity phosphatase 6	NM_053883	Rn00518185_m1
Fmr1	Fragile X mental retardation syndrome 1 homolog	NM_052804	Rn00709627_m1
Glud1	Glutamate dehydrogenase 1	NM_012570	Rn00561306_m1
Gria2	Glutamate receptor, ionotropic, AMPA2	NM_017261	Rn00568514_m1
Hif1a	Hypoxia inducible factor 1, alpha subunit	NM_024359	Rn00577560_m1
Myst2	MYST histone acetyltransferase 2	NM_181081	Rn00710321_m1
Npy	Neuropeptide Y	NM_012614	Hs00173470_m1
Npy5r	Neuropeptide Y receptor Y5	NM_012869	Rn02089867_s1
Slit2	Slit homolog 2	NM_022632	Rn00575268_m1
Rgs2	Regulator of G-protein signaling 2	NM_053453	Rn00584932_m1
Wasl	Wiskott-Aldrich syndrome-like (human)	NM_001034130	Rn01501132_m1

### Ontological, pathway, and network analysis

Ontological analysis used Gene Ontology (GO) categories to determine processes or functional categories that were differentially expressed, as described previously [32] using GeneSpring GX software. This analysis determined the number of genes in a category present on the array and the number of expression changes that would be part of that category by random chance given the number of differentially expressed genes. Ingenuity Pathway Analysis (Ingenuity Systems, Redwood City, CA) was used to create a network from RT-qPCR confirmed gene expression results from the rats described in this study.

### Statistical Analysis

Behavioral data were analyzed by t-tests at each time point of abstinence (1 d and 14 d). Goal-directed behavior was determined by subtracting inactive spout responses from active spout responses. RT-qPCR gene expression values were evaluated using t-tests between self-administering and yoked saline rats at each time point of abstinence (1 or 14 d). For both behavioral and gene expression data, levels of significance were determined with α set at 0.05. Correlational analyses were performed to determine whether a correlation between the goal-directed behavior or active spout responses during the extinction session and gene expression existed.

## Results

### Behavior

Rats were allowed to self-administer heroin during 14 days of daily drug access. The average daily heroin intake for the rats that experienced only 1 day of abstinence prior to extinction increased from 7.3 ± 0.5 infusions on day 1 to 12.0 ± 2.5 infusions on day 14 of self-administration. Average daily heroin intake for the group of rats that experienced 14 days of abstinence prior to extinction increased from 7.2 ± 0.6 infusions on day 1 to 10.1 ± 3.1 on day 14 of self-administration. When rats were reintroduced to the self-administration chambers following 1 d or 14 d of abstinence, incubation of active spout responses was observed (defined as a significant increase in responses on the active spout with the progression of time [21]. Goal-directed behavior also incubated in self-administering rats and increased from an average of 80.6 +/- 42.5 responses following 1 day of abstinence to an average of 302.6 +/- 45.9 responses following 14 days of abstinence (Figure 1). Goal-directed behavior after 14 days of abstinence was significantly higher in self-administering vs. yoked saline rats (p < 0.001). Additionally, 14 day abstinent heroin self-administering rats had higher goal-directed behavior than self-administering rats with only 1 day of abstinence (p < 0.01).

### Microarray analysis

Signals from 23,670 probes (of the > 41,000 total probes) were detected as being present on all of the arrays. Filtering the detected genes produced a list of 66 genes that were identified as being changed by at least 1.4 fold (at the p < 0.02 level of significance) relative to saline controls after 14 days of abstinence and a 90-minute extinction session. A full listing of differentially expressed genes is provided in Additional File 1.

### RT-qPCR Confirmation and Validation

RT-qPCR was performed to confirm expression levels of genes for which significant differences in expression were detected by microarrays. Genes were chosen for confirmation analyses based on their ontological classifications and probable involvement in drug use. Many genes examined belonged to ontological categories of nervous system development or behavior. For each gene examined, samples from self-administering and yoked saline rats from each of the abstinent period treatment groups (1 day and 14 days) were examined.

Table 1 depicts the 19 genes on which RT-qPCR was performed for this study. Additionally, Nr4a3 was detected by our arrays to be significantly increased and has been previously reported to be increased by another laboratory [33] and EGR1 was confirmed to be changed in our previous report of gene expression changes [22]. Of the genes tested in the present study, 6 (*bdnf, calb1, dusp5, dusp6, npy, rgs2*) were validated by RT-qPCR to be changed at a significance level of at least α = 0.05. The other 13 genes listed in Table 1 were identified by the microarray to have significant differences in gene expression, but the RT-qPCR results did not validate expression differences for these genes. Reasons for why all 19 genes were not detected as changed by RT-qPCR include the fact that many of these genes are expressed near the levels of detection of the array platform. In addition, false positive results are common to arrays and account for the need to provide post-hoc confirmations.

**Figure 2:**
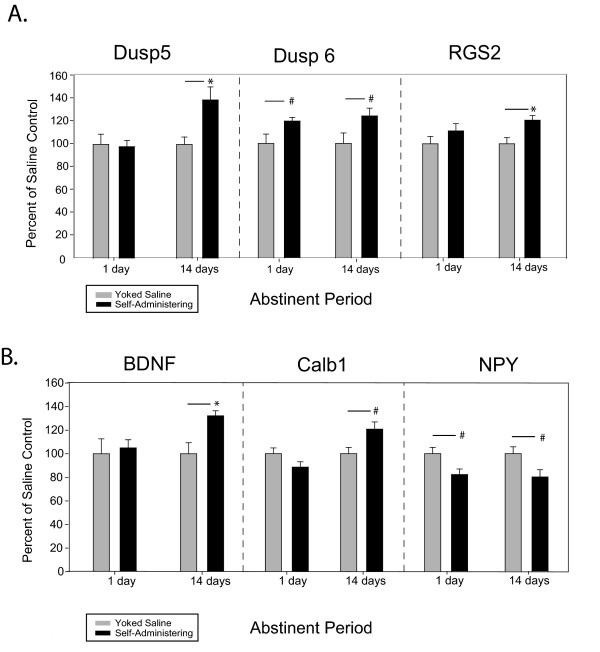
**A. Genes involved in regulation of intracellular signaling**. RT-qPCR confirmed gene expression changes in self-administering compared to yoked saline rats after 1 and 14 days of abstinence. B. Genes involved in neuronal adaptations to behavior. RT-qPCR confirmed gene expression changes in self-administering compared to yoked saline rats after 1 and 14 days of abstinence. In both panels, data represent means ± SEM. * p < 0.01, # p < 0.05.

Among the genes that were detected by the arrays to be significantly changed, and confirmed by RT-qPCR, were genes that are important for intracellular signaling (Figure 2A). Dual specificity phosphatase 5 (*dusp5*) and dual specificity phosphatase 6 (*dusp6*) expression levels were both increased by 39% and 24%, respectively, after 14 days of abstinence, although only *dusp6 *expression levels were increased after only 1 day of abstinence. Regulator of G-protein signaling 2 (*rgs2*) expression was increased by 20% following 14 days of abstinence.

Genes for additional intracellular molecules that each can be linked to physiological changes that occur following drug use were also confirmed to be significantly changed (Figure 2B). Brain-derived neurotrophic factor (*bdnf*) and calbindin 1 (*calb1*) both displayed increased expression levels, by 32% and 21%, respectively, relative to saline controls. Neuropeptide Y (*npy*) gene expression (tested because the array suggested that expression of one of its receptors - npy5r - was altered) was decreased by 18% at 1 day of abstinence and 20% at 14 days of abstinence.

### Ontological, pathway, and network analysis

Analysis of microarray gene expression data by gene ontology revealed 24 genes that are involved in developmental processes. Of these genes, 4 were confirmed to be changed by RT-qPCR: *bdnf, calb1, dusp6, egr1 *and one (*nr4a3*) has been previously reported by another group of researchers [33]. A second ontological category of interest was behavior, and 17 genes from our list were included in this category. Among these genes were *egr1 *and *crybb1*.

**Figure 3:**
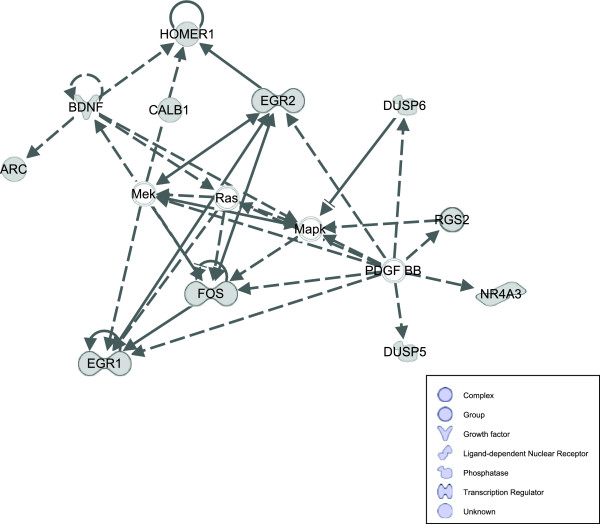
**Ingenuity pathway analysis**. Gene expression changes that have been confirmed function together in a network that is important for nervous system development. Direct relationships are indicated by solid lines while indirect relationships are indicated by dashed lines.

The Ingenuity network analysis revealed that many genes validated in this study, together with genes previously reported from these samples to be significantly changed, can be linked through a hypothetical network that is involved with behavior, nervous system development and function, and cellular development (Figure 3). The pathway depicted in this figure is based on known intermolecular relationships (but not necessarily direct physical interactions), although whether this entire network of interactions occurs *in vivo *remains to be elucidated. All of the genes in this pathway were determined to be upregulated.

### Correlational Analyses

Behavioral data collected during extinction (goal-directed responses and active spout responses) was used in correlational analyses with gene expression data. Both Pearson's and Spearman's correlations were performed because of the small sample sizes that were not always normally distributed. When separate correlations were performed for data from each time point, Pearson's analyses identified a positive correlation between the number of active spout responses and *dusp5 *gene expression in rats that experienced 1 day of abstinence (p = 0.05; r = 0.70, data not shown). A significant negative correlation between *dusp5 *gene expression and inactive spout responses (p < 0.03; r = -0.78) was detected for rats that experienced 14 days of abstinence by a Spearman's analysis. Combining the data from both abstinent periods yielded 8 more significant correlations (Table 2).

**Table 2:** Correlational analysis results

	Pearson				Spearman			
	
	BDNF	Calb1	Dusp5	NPY	BDNF	Calb1	Dusp5	NPY
Active Spout Responses	0.58	0.10	0.37	-0.20	0.59	0.08	0.42	-0.18
	0.02*	0.73	0.18	0.49	0.02*	0.78	0.11	0.52

Goal-Directed Behavior	0.64	0.48	0.61	-0.55	0.65	0.53	0.48	-0.58
	0.01*	0.07	0.02*	0.03*	0.009*	0.04*	0.07	0.02*

Goal-directed response and active spout response data from extinction were correlated with RT-qPCR data. Eight significant correlations were detected (4 detected by Pearson's analysis and 4 detected by Spearman's analysis). The upper number in each block of the table is the correlation coefficient and the lower number in each block of the table is the P value.

## Discussion

This study provides the first report of whole genome analysis of mPFC gene expression in rats that have expressed behavioral incubation and expands our knowledge of gene expression changes that exist after abstinence and during the time of relapse liability. The approach of using a preliminary screen of the entire genome, followed by rigorous RT-qPCR confirmation of expression changes for genes likely to be involved in behavior or neuronal changes has yielded information on genes, such as *dusp5 *and *dusp6*, whose role in addiction is only now beginning to emerge.

The majority of genes that were significantly changed using the criteria reported in this paper (52 of 66 genes) had increased expression levels in heroin self-administering rats when compared to yoked saline following 14 days of enforced abstinence. Previously, increased gene expression has been found for opiate receptors in humans that died from an opiate overdose [34], as well as components of the cyclic AMP signaling system following opiate use [35]. The reasons for a disproportionate number of up-regulated versus down-regulated genes following abstinence from heroin self-administration remain to be determined. However, when gene expression nears the level of detection of the microarray platform (as many of these did), it is becomes more difficult to detect down-regulations.

For the genes identified, and confirmed in this study, there are several potential impacts on behavior. Phosphatase genes, such as *dusp5 *and *dusp6 *(MKP-3; MAP kinase phosphatase-3), encode proteins that have a direct impact on intracellular signaling. *Dusp5 *preferentially dephosphorylates ERK [36,37] and is intranuclear, while *dusp6 *is cytoplasmic [38]. MDMA (3,4-methylenedioxymethamphetamine) has been shown to increase expression of *dusp 1*, *dusp 5*, and *dusp 14 *genes [39], while methamphetamine treatment increases *dusp6 *gene expression in multiple brain regions [40]. Both *dusp5 *[41] and *dusp6 *[42] regulate mitogen-activated protein kinase (MAPK). Numerous studies have suggested MAPK involvement in neuroadaptations that occur following drug use [43-45]. Beyond being affected by mere drug exposure, MAPK molecules have also been shown to play a role in morphine tolerance [46] and to be activated following opiate withdrawal [47]. Interestingly, the MAPK pathway plays a central role in the relationships between genes that were reported as changed in this study and our previous study of gene expression (Figure 3; [22]).

Molecules that regulate G-protein coupled receptor (GPCR) signaling have been shown to be essential for reinstatement of heroin-seeking behavior [48]. The observed increase in *rgs2 *gene expression following 14 days of abstinence and contextual re-exposure may represent an intracellular signaling change that affects communication between receptors and transcription factors, ultimately affecting cellular and organismal physiology. A GPCR-regulating molecule (AGS3; Activator of G-protein signaling 3) was shown, through a gene knock-down approach, to be essential to activating protein kinase A (PKA) signaling and observing reinstatement of heroin-seeking [48].

The microarray analysis detected an NPY receptor, NPY5R, to be significantly increased after 14 days of abstinence. While RT-qPCR for *npy5r *failed to replicate this change, the gene encoding *npy*, the ligand for this receptor, was tested by RT-qPCR and found to be changed. *npy *gene expression differed from most other genes examined because it was decreased in rats that had self-administered heroin, both after 1 and 14 days of abstinence. Although *npy *is a neurotransmitter most recognized for its role in regulating food intake [49], it has been hypothesized that *npy *may contribute to the negative motivational state of withdrawal [50]. Intracerebroventricular administration of *npy *has been found to block increased ethanol intake in rats [50,51], and overexpression of the *npy *gene, using intra-amygdalar infusions of a viral expression vector, diminished alcohol intake following longer periods of abstinence or repeated alcohol withdrawals [52]. In the present context, intraventricular injections of *npy *have been found to induce heroin-seeking behavior following extinction sessions [53], suggesting that the decrease in *npy *gene expression observed in the present study may be related to the extinction component of the behavioral testing.

The majority of genes on which RT-qPCR was performed displayed expression levels significantly different from saline controls only after 14 days of abstinence (*dusp5, rgs2, bdnf, calb1*). This suggests that these changes are attributable either solely to the extended drug abstinence or to the combination of extended abstinence and contextual re-exposure. It is documented that exposure to environments previously paired with drug administration can affect gene expression [54-56], so future studies will be required to discern whether the observed changes in gene expression after 14 days of abstinence resulted from contextual re-exposure, the pharmacological drug abstinence, or a combination. Correlational analyses detected that *bdnf *was significantly correlated with both goal-directed behavior and active spout responses (Table 2). This observation supports the proposed importance of *bdnf *to drug-seeking behavior [57]. While the study was not originally powered to conduct individual correlational analyses, the findings with *bdnf *suggest that future work may focus on individual behavior and gene expression.

Several genes whose expression was changed in this study have also displayed changed expression levels following several extinction sessions [33]. In the mPFC, there was an increase in gene expression relative to controls for *arc*, *homer1a*, *ania-3*, *mkp-1*, *c-fos*, *egr1*, *egr2*, and *nr4a3*. *homer1*, *egr1*, and *nr4a3 *each were detected by the arrays to have increased expression values of at least 1.4-fold in the current study. The repeated extinction sessions in the Koya et al. study [33] are a major difference from our study that may contribute to the differences in gene expression observed between studies. Genes that were changed in both Koya's study, which included 14 extinction sessions, and the present study, which included 1 extinction session, are genes that apparently exhibit changed expression following a prolonged abstinence and maintain that change in expression regardless of the amount of environmental re-exposures.

The identities of the confirmed genes include not only transcription factors, but also genes for molecules involved in intracellular signaling and protein binding (Figure 3). This range of functions (transcription factors, receptors, ion channels) provides a reminder that drug use elicits changes in entire intracellular networks. The importance of changes in gene expression of certain proteins, such as *calb1 *or *dusp6*, to eliciting changed expression of genes encoding transcription factors, such as *egr1 *or *fos*, is an area for future investigation.

While this microarray analysis has illuminated genes whose expression is changed immediately following an extinction session, the gene expression profile that exists prior to extinction testing has not been examined in this study. A comparison between the gene expression profile following abstinence versus the gene expression profile following abstinence and extinction would address the question of what expression changes are specifically induced by the extinction behavior itself. The increased activity expressed after 14 days of abstinence may, in part, have been the cause, rather than the result, of observed gene expression changes. However, such changes would fall into the category of immediate early changes as the extinction testing was only 90 minutes in duration. The value of the present study is that it focuses on a time point at which the subjects have experienced contextual re-exposure, a key component for eliciting relapse. This study has produced data that are relevant not only for future studies involving heroin, but also for understanding the molecular underpinnings of incubation of drug-seeking.

## Conclusion

We have identified a group of genes whose expression is significantly changed following abstinence from heroin self-administration and incubation of heroin-seeking behavior. Confirmed genes are not limited to genes encoding transcription factors, but also encompass genes encoding molecules that are important for regulation of intracellular signaling. These regulatory molecules may be effective targets for drug interventions to prevent relapse.

## Authors' contributions

KLK performed behavioral procedures, data analysis, and drafted the manuscript. RMB performed the microarray experimental procedures. PSG contributed to the behavioral design. WMF contributed to the design and interpretation of the microarray experiment. KEV participated in the design and coordination of the study. All authors made contributions to the final manuscript.

## Supplementary Material

Additional file 1 **Changed genes on array**. The data provided are the complete list of 66 genes that were identified to have changed expression at the p < 0.02 level of significance. Click here for file
